# From Sea to Cell: *Ascophyllum nodosum* and *Fucus vesiculosus* Extracts Attenuate NF-κB-Mediated Inflammation and Protect Intestinal Barrier Integrity—A Comprehensive Analysis Applying In Vitro and In Vivo Models

**DOI:** 10.3390/md24050182

**Published:** 2026-05-19

**Authors:** Lea Karlsberger, Nadiia Sadova, Mara Heckmann, Fanny Serenius, Annika Meinander, Julia Kirchsteiger, Alice König, Bettina Schwarzinger, Bernhard Blank-Landeshammer, Stephanie Ladirat, Julian Weghuber

**Affiliations:** 1 FFoQSI GmbH, Austrian Competence Centre for Feed and Food Quality, Safety and Innovation, Technopark 1D, 3430 Tulln, Austria; lea.karlsberger@fh-wels.at (L.K.); nadiia.sadova@fh-wels.at (N.S.); mara.heckmann@fh-wels.at (M.H.); julia.kirchsteiger@fh-wels.at (J.K.); bettina.schwarzinger@fh-wels.at (B.S.); 2 Center of Excellence Food Technology and Nutrition, University of Applied Sciences Upper Austria, Stelzhamerstraße 23, 4600 Wels, Austria; alice.koenig@fh-wels.at (A.K.); bernhard.blank-landeshammer@fh-wels.at (B.B.-L.); 3 Faculty of Science and Engineering, Biochemistry and Cell Biology, Åbo Akademi University, Henrikinkatu 2, 20500 Turku, Finland; fanny.serenius@abo.fi (F.S.); annika.meinander@abo.fi (A.M.); 4 InFLAMES Research Flagship Center, Åbo Akademi University, Henrikinkatu 2, 20500 Turku, Finland; 5 SAS NUQO, Chez Axes Compta Conseils, Immeuble l’ONYX, 28 Avenue du Pamelan, 74000 Annecy, France; ladirat.stephanie@nuqo.eu

**Keywords:** brown algae, *Ascophyllum nodosum*, *Fucus vesiculosus*, inflammatory signaling, NF-κB, *Drosophila melanogaster*

## Abstract

The restriction of antimicrobial growth promoters in livestock production has intensified the search for nutritional strategies that support intestinal health while modulating inflammatory processes. Chronic or dysregulated inflammation can impair gut function and animal performance, highlighting the need for functional feed additives. Brown macroalgae are rich in bioactive compounds with immunomodulatory properties, though their mechanisms remain incompletely understood. In this study, the anti-inflammatory and barrier-protective effects of aqueous extracts from *Ascophyllum nodosum* (AN) and *Fucus vesiculosus* (FV) were investigated using complementary in vitro and in vivo models. Extracts were prepared by aqueous solid–liquid extraction and tested in lipopolysaccharide (LPS)-stimulated RAW264.7 and THP-1 macrophages, HEK-Blue TLR4 reporter cells, and *Drosophila melanogaster* models of intestinal inflammation and infection. Both extracts significantly reduced LPS-induced nitric oxide production in RAW264.7 macrophages in a concentration-dependent manner. In THP-1 macrophages, AN and FV attenuated secretion of inflammatory mediators, including TNF-α, IL-6, IL-33, CXCL9, CXCL10, CXCL11, and CCL7. Reporter assays demonstrated selective inhibition of TLR4-dependent NF-κB activation. In *Drosophila melanogaster*, supplementation reduced intestinal barrier disruption, mortality, and infection-induced immune activation. Overall, AN and FV attenuate inflammatory signaling and protect intestinal integrity via TLR4-dependent NF-κB inhibition, supporting their potential as functional feed additives to enhance gut health and resilience in livestock.

## 1. Introduction

The restriction and banning of antimicrobial growth promoters (AGP) in animal production, particularly within the European Union, has been driven by increasing public health concerns regarding antimicrobial resistance. While the removal of AGP has successfully reduced the prevalence of resistant bacterial populations, it has also been associated with a higher incidence of enteric disorders and inflammatory challenges, negatively affecting animal health, welfare, and production efficiency. Consequently, there is an urgent need for nutritional strategies that maintain gut health and immune homeostasis without reliance on antibiotics [1,2,3].

Intestinal health is a multidimensional concept encompassing the integrity of the epithelial barrier, the function of the mucosal immune system, the composition and activity of the resident microbiota, and the interactions with dietary components. Disruption of this equilibrium often leads to inflammation, impaired barrier function, reduced nutrient absorption, and increased susceptibility to pathogens. Even subclinical or low-grade inflammation imposes a considerable metabolic cost, as nutrients are redirected from growth and production toward immune defense [4,5]. Maintaining gut homeostasis is therefore essential not only for animal welfare but also for sustainable production efficiency.

At the molecular level, intestinal inflammation is initiated through the activation of pattern recognition receptors (PRRs), including toll-like receptors (TLRs), expressed by epithelial and immune cells. Engagement of these receptors activates intracellular signaling cascades that converge on nuclear factor kappa B (NF-κB), a central transcription factor regulating the expression of pro-inflammatory cytokines, chemokines, and other mediators of the inflammatory response. While acute activation of these pathways is essential for host defense, persistent or excessive activation contributes to chronic inflammation, tissue damage, and impaired intestinal barrier integrity, ultimately compromising animal health and performance [1,6,7].

Oxidative stress is closely linked to intestinal inflammation. Immune activation is accompanied by the production of reactive oxygen species (ROS), which are required for pathogen defense but can cause cellular damage when generated in excess. Excessive ROS further amplifies inflammatory signaling through redox-sensitive pathways, including NF-κB, creating a self-perpetuating cycle of inflammation and tissue injury [8,9]. This interplay between oxidative stress and inflammation is increasingly recognized as a key factor underlying reduced resilience and productivity in production animals [1].

Plant- and algae-derived feed additives containing bioactive secondary metabolites have gained attention as potential alternatives to AGP. In addition to algae-derived compounds, several other non-antibiotic nutritional strategies, including probiotics, prebiotics, synbiotics, organic acids, yeast-derived products, and phytogenic feed additives, have been investigated to support gut health, immune homeostasis, and production performance in livestock [10,11,12]. Among these alternatives, brown macroalgae are of particular interest due to their high content of bioactive compounds with antioxidant and immunomodulatory properties, including polyphenols and complex polysaccharides [13,14]. Supplementation with brown algae or their extracts has been shown to improve antioxidant status, enhance immune responses, modulate the gut environment, and support production performance in pigs and poultry [15,16,17]. However, mechanistic evidence elucidating how these extracts influence key immune signaling pathways and protect gut barrier function remains limited.

The biological activity of brown algae is generally attributed to their diverse bioactive metabolites. Among these, phlorotannins, a group of polyphenolic compounds unique to brown macroalgae, have been widely reported to possess antioxidant and anti-inflammatory activities. Certain phlorotannins can modulate inflammatory signaling pathways, including inhibition of NF-κB activation and reduction of pro-inflammatory mediator production in cellular models [18,19]. In addition, brown algae contain fucoidans, sulfated fucose-rich polysaccharides abundant in species such as *Ascophyllum nodosum* and *Fucus vesiculosus.* Fucoidans exhibit immunomodulatory and anti-inflammatory properties and, due to their resistance to digestion in the upper gastrointestinal tract, may also interact with the intestinal microbiota [20,21]. These polyphenolic and polysaccharide components are therefore considered key contributors to the biological effects associated with brown algae and represent promising candidates for functional feed additives aimed at supporting intestinal health in livestock.

Despite growing evidence of bioactivity, the in vivo effects of algal compounds on intestinal immune signaling and epithelial barrier function have not been extensively characterized. Experimental model systems that allow for controlled investigation of host–microbe interactions and conserved inflammatory pathways are therefore valuable for mechanistic studies of dietary bioactives.

*Drosophila melanogaster* has emerged as a valuable in vivo model for studying gut health and dietary interventions. Although an invertebrate, *D. melanogaster* shares key elements of innate immunity with mammals, such as conserved signaling pathways that regulate antimicrobial responses and gut defense. In flies, the Toll and immune deficiency (Imd) pathways activate NF-κB-like transcription factors to induce antimicrobial peptides in response to microbial signals, providing a tractable system to investigate innate immune mechanisms relevant across species [22]. The fly intestine comprises a compartmentalized epithelium with barrier functions and dynamic host–microbe interactions resembling aspects of vertebrate gut physiology [23]. Its short lifespan, genetic tractability, and well-characterized microbiota allow rapid, high-throughput assessment of bioactive compounds on intestinal barrier integrity and immune function [24]. Using this model, it is possible to evaluate both preventive and protective effects of dietary interventions against chemical or bacterial intestinal challenges, providing mechanistic insights that inform subsequent studies in vertebrate systems.

Among brown algae, *A. nodosum* and *F. vesiculosus* are extensively studied for animal nutrition. In a previous study, we demonstrated the pronounced antioxidant activity of aqueous extracts from these species in intestinal epithelial cells and in *C. elegans* under oxidative challenge [25]. Building on these findings, the present study investigates their anti-inflammatory potential in lipopolysaccharide (LPS)-stimulated macrophage models, their modulation of NF-κB signaling, and their ability to protect intestinal barrier integrity in *D. melanogaster*. This integrated approach provides mechanistic and translational insight into algae-derived dietary strategies for maintaining gut health and immune homeostasis in production animals.

## 2. Results

### 2.1. Chemical Characterization of AN and FV Extracts

The chemical composition of aqueous extracts from the brown algae *A. nodosum* (AN) and *F. vesiculosus* (FV) has been previously characterized [25] and is briefly summarized here to facilitate interpretation of the biological findings. Extracts were analyzed using photometric assays and LC-HRMS/MS. Total phenolic content (TPC), antioxidant capacity (TEAC and FRAP), and total polysaccharide content (TPSC) were quantified, and phlorotannins were tentatively identified based on accurate mass and fragmentation patterns.

FV exhibited substantially higher phenolic content and antioxidant capacity com-pared to AN, with approximately threefold higher TPC, TEAC, and FRAP values (Table S1). In contrast, AN contained higher level of total polysaccharides (71 vs. 48 mg glucose equivalents/g dry weight).

LC-HRMS/MS analysis revealed the presence of phlorotannins with varying degrees of polymerization (DP 2–9) in both extracts (Tables S2 and S3). FV showed a broader structural diversity and higher molecular weight phlorotannins, including oligomers with DP ≥ 8 that were not detected in AN. In contrast, AN contained a smaller set of lower molecular weight phlorotannins (DP 2–7).

Overall, FV is characterized by a higher abundance and diversity of phenolic compounds, particularly high-molecular-weight phlorotannins, whereas AN is enriched in polysaccharides.

### 2.2. AN and FV Reduce the Nitric Oxide Production in LPS-Stressed RAW264.7 Cells

The anti-inflammatory potential of AN and FV was first assessed by evaluating their effects on nitric oxide (NO) production under LPS-induced inflammatory conditions. Stimulation of RAW264.7 macrophages with LPS induces increased NO production, a key mediator in inflammatory signaling [26].

**Figure 1:**
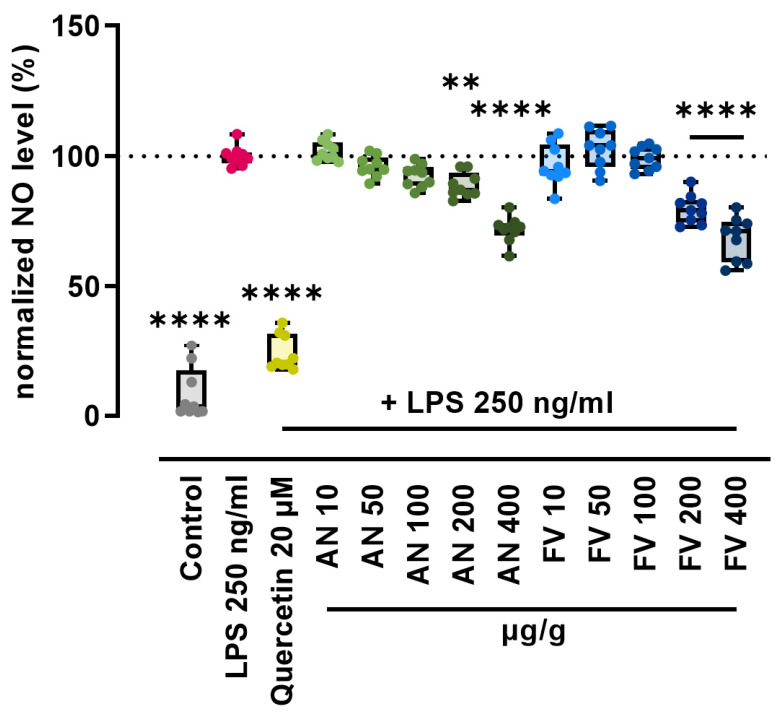
AN and FV reduce inflammatory NO production in LPS-challenged RAW264.7 cells. RAW264.7 cells were co-treated with LPS (250 ng/mL) and AN, FV, or quercetin (20 µM) for 24 h. Nitrite levels in culture supernatants were quantified using the Griess Reagent System. Absorbance was measured at 548 nm, and nitrite concentrations were normalized to the LPS control. The dotted line at 100% indicates the normalized LPS control and serves as a visual reference baseline for comparison between groups. Statistical analysis was performed using one-way ANOVA followed by Dunnett’s multiple comparisons test. Data are presented as median with interquartile range (IQR), whiskers indicating minimum and maximum values, and individual data points from three independent experiments (*n* = 9). Statistical significance is indicated as ** *p* ≤ 0.01 and **** *p* ≤ 0.0001.

RAW264.7 cells were co-treated with LPS and AN or FV at concentrations of 10, 50, 100, 200, and 400 µg/g for 24 h. Quercetin (20 µM), a well-characterized flavonoid with established antioxidant and anti-inflammatory activity known to suppress LPS-induced NO production in macrophages, was used as a positive control [27]. NO levels in the culture supernatant were quantified using the Griess Reagent System. Normalized NO concentrations are presented in Figure 1.

LPS stimulation significantly increased NO production in RAW264.7 macrophages compared with untreated controls. Treatment with quercetin markedly attenuated LPS-induced NO production (*p* ≤ 0.0001). Both AN and FV significantly reduced NO levels in a concentration-dependent manner, with significant effects observed at concentrations ≥ 200 µg/g (AN: *p* ≤ 0.01; FV: *p* ≤ 0.0001). At 200 and 400 µg/g, AN reduced NO production to 0.89 and 0.72 relative to the LPS control, corresponding to reductions of approximately 11% and 28%, respectively. In contrast, FV reduced NO levels to 0.80 and 0.68 at the same concentrations, representing reductions of approximately 20% and 32%. At equivalent concentrations, FV consistently elicited a greater inhibition of NO production than AN, indicating a stronger attenuation of nitrosative stress under the conditions tested.

### 2.3. AN and FV Influence the Production of Cytokines and Chemokines Under LPS-Challenge

To assess the impact of AN and FV on inflammatory signaling, a semi-quantitative cytokine array analysis was performed using LPS-stimulated THP-1 macrophages. Cells were exposed to LPS to induce the production of pro-inflammatory mediators and co-treated with AN or FV at 400 µg/g. Culture supernatants were incubated on membranes pre-coated with antibodies against 105 human cytokines and chemokines, and analyte levels were evaluated based on visualized spot intensity.

**Figure 2:**
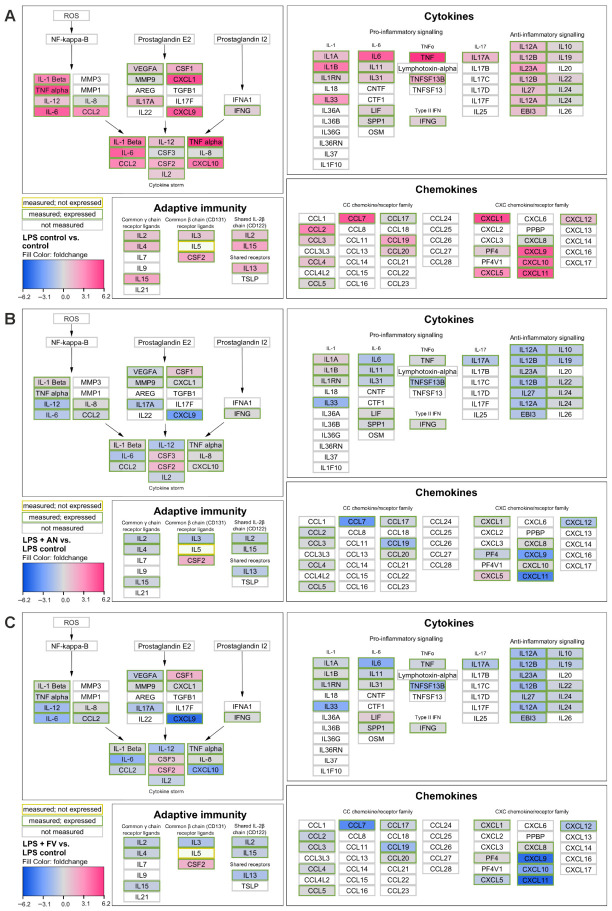
Overview of cytokine and chemokine expression in LPS-stimulated THP-1 macrophages following treatment with AN or FV. Cytokine and chemokine expression patterns were assessed by semi-quantitative cytokine array analysis and visualized as fold changes in analyte levels. (**A**) Comparison of LPS-treated cells with untreated control cells. (**B**) Comparison of LPS stimulation alone with LPS stimulation in the presence of AN (400 µg/g). (**C**) Comparison of LPS stimulation alone with LPS stimulation in the presence of FV (400 µg/g). Cytokines and chemokines outlined in gray were not included in the analysis, those outlined in yellow were measured but not detected in THP-1 cells, and those outlined in green were both measured and expressed. Color-filled nodes indicate regulated mediators, with blue representing decreased and pink increased expression. Data represent mean values from one experiment (*n* = 2).

Overall cytokine expression patterns are summarized as fold changes relative to untreated control cells (LPS vs. control; Figure 2A) or relative to LPS stimulation alone in the presence of AN (Figure 2B) or FV (Figure 2C). LPS treatment induced a broad increase in the production of pro-inflammatory cytokines and chemokines associated with NF-κB-related signaling, as well as prostaglandin E2 and I2 pathways, reflecting a pronounced pro-inflammatory activation state.

Co-treatment with AN or FV attenuated this inflammatory profile by reducing the production of a comparable set of cytokines and chemokines relative to LPS alone. Regulated analytes are displayed using a color gradient, with downregulation shown in blue and upregulation in pink. While AN and FV affected largely overlapping cytokine profiles, differences were observed in the extent of downregulation, with FV generally exerting a stronger suppressive effect than AN.

**Figure 3:**
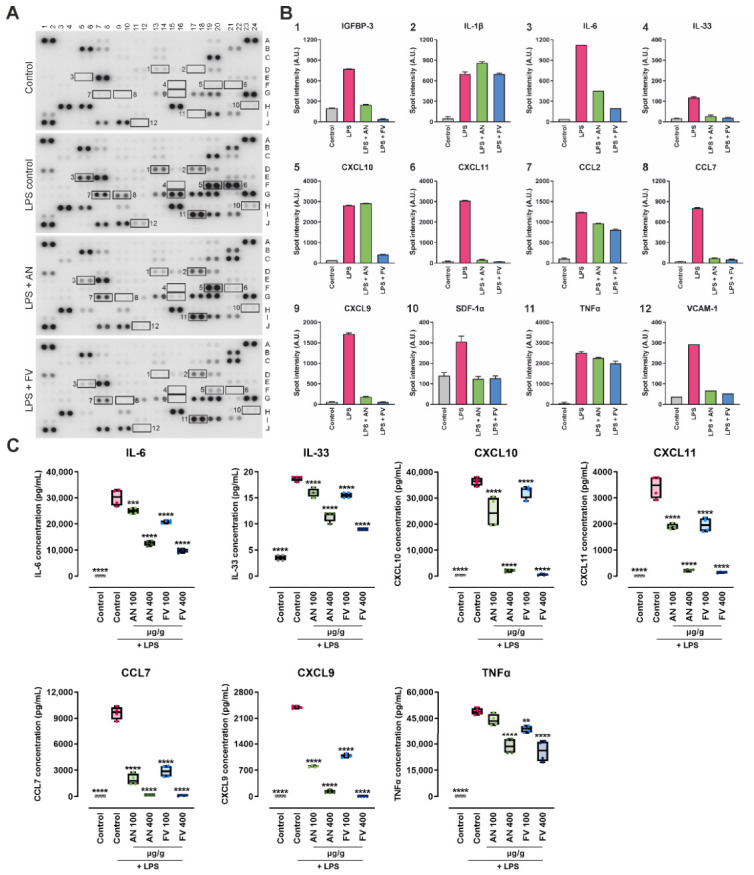
Effects of AN and FV on cytokine and chemokine expression in LPS-stimulated THP-1 macrophages. (**A**) Representative cytokine array membranes incubated with culture supernatants from THP-1 cells stimulated with LPS and co-treated with AN or FV (400 µg/g). Expressed cytokines and chemokines are detected as duplicate black spots on the membrane; selected analytes of interest are highlighted and numbered. (**B**) Quantification of the corresponding spot intensities for the indicated analytes, presented as arbitrary units (A.U.). (**C**) Concentrations of IL-6, IL-33, CXCL10, CXCL11, CCL7, CXCL9, and TNF-α determined using a multiplex bead-based immunoassay. Statistical analysis was performed using one-way ANOVA followed by Dunnett’s multiple comparisons test. Data are shown as median with interquartile range (IQR), whiskers (min/max values), and individual data points from two independent experiments (*n* = 4). Statistical significance is indicated as ** *p* < 0.01, *** *p* < 0.001, and **** *p* < 0.0001, with asterisks referring to comparisons against the LPS-stressed control group.

Representative cytokine array membranes are shown in Figure 3A, highlighting selected analytes that were induced upon LPS challenge compared to control conditions. The corresponding spot intensity values are presented in Figure 3B. Both AN and FV reduced the LPS-induced release of key inflammatory mediators, including IGFBP-3, IL-6, IL-33, CXCL9, CXCL11, CCL2, CCL7, SDF-1α, TNF-α, and VCAM-1. For these mediators, FV consistently produced a greater reduction in signal intensity than AN, indicating a stronger inhibition of inflammatory mediator production rather than differences in pathway selectivity.

To further quantify selected cytokines and chemokines identified in the array analysis, concentrations of TNF-α, IL-6, IL-33, CCL7, CXCL9, CXCL10, and CXCL11 were measured in the supernatants of THP-1 macrophages treated with AN or FV at two concentrations (100 µg/g and 400 µg/g) using a bead-based multiplex immunoassay (Figure 3C). Consistent with the semi-quantitative cytokine array results, AN treatment led to significant, concentration-dependent reductions in multiple inflammatory mediators. IL-6 levels were significantly reduced at both 100 µg/g (*p* = 0.0006) and 400 µg/g (*p* < 0.0001). Similarly, significant decreases in IL-33, CXCL10, CXCL11, CCL7, and CXCL9 were observed at both AN concentrations tested (*p* < 0.0001). A significant reduction in TNF-α levels was detected following treatment with 400 µg/g AN (*p* < 0.0001), whereas the lower concentration had a more limited, non-significant effect. FV treatment resulted in a significant reduction in all measured cytokines and chemokines at both concentrations. For most analytes, this effect was highly pronounced (*p* < 0.0001), with the exception of TNF-α at 100 µg/g FV, which reached a lower level of statistical significance (*p* = 0.0035).

At equivalent concentrations, FV generally exerted stronger suppressive effects on IL-6, IL-33, and TNF-α levels compared to AN, indicating a higher efficacy of FV in reducing these mediators. In contrast, for the chemokines CXCL10, CXCL9, CCL7, and CXCL11, treatment with 100 µg/g AN resulted in a greater reduction than the corresponding FV concentration. However, at the higher dose of 400 µg/g, FV produced a more pronounced decrease in these chemokines than AN, suggesting distinct dose–response characteristics between the two treatments.

### 2.4. AN and FV Inhibit NF-κB Activation in HEK-Blue Reporter Cell Lines

To assess whether the anti-inflammatory effects of AN and FV are mediated via TLR4-dependent NF-κB signaling, NF-κB activation was analyzed using the HEK-Blue hTLR4 reporter cell line and its corresponding control cell line, HEK-Blue null2. HEK-Blue hTLR4 cells stably express human TLR4 along with a SEAP reporter gene under the control of NF-κB, whereas HEK-Blue null2 cells lack TLR4 expression but retain NF-κB-driven SEAP production. Accordingly, SEAP activity measured in the culture supernatant directly reflects NF-κB activation.

**Figure 4:**
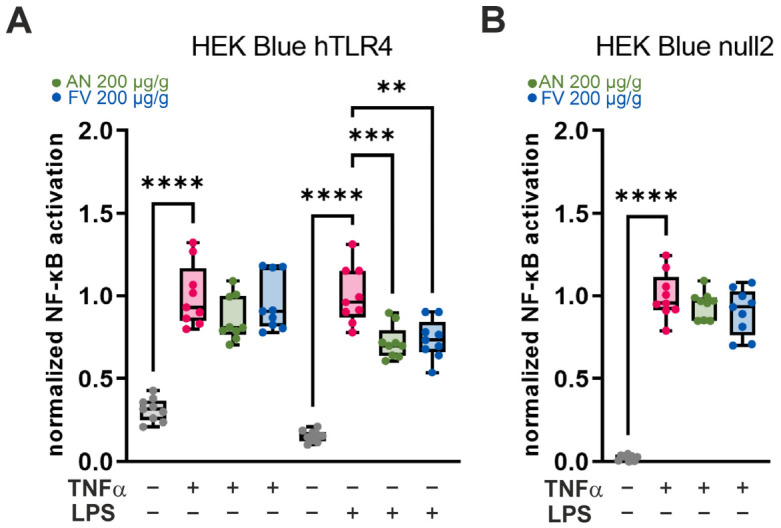
Effects of AN and FV on NF-κB activation in reporter cell lines. NF-κB activation was determined in (**A**) non-TLR4-expressing HEK-Blue null2 cells and (**B**) TLR4-expressing HEK-Blue hTLR4 cells following 24 h of stimulation with TNF-α (10 ng/mL) or LPS (10 ng/mL) and AN or FV (200 µg/g). NF-κB activity was quantified by SEAP levels in the culture supernatant and normalized to the respective stressor control. Statistical analysis: one-way ANOVA with Tukey’s multiple comparison test. Data are presented as median with interquartile range (IQR), whiskers (min/max values), and individual data points from three independent experiments (*n* = 9). Significant *p* values are indicated as ** (*p* ≤ 0.01), *** (*p* ≤ 0.001), **** (*p* ≤ 0.0001).

Figure 4 shows the NF-κB activation measured in the supernatant of HEK-Blue hTLR4 and HEK-Blue null2 cells after treatment with TNF-α or LPS and AN or FV normalized to the respective stressor. Stimulation of HEK-Blue hTLR4 cells with LPS or TNF-α resulted in a significant induction of NF-κB activity compared with unstimulated control cells (both *p* < 0.0001). In contrast, HEK-Blue null2 cells responded to TNF-α stimulation with a significant increase in NF-κB activation (*p* < 0.0001), while LPS treatment did not induce NF-κB signaling, confirming the absence of functional TLR4 signaling in these cells.

Treatment with AN or FV at a concentration of 200 µg/g did not significantly affect NF-κB activation in HEK-Blue null2 cells, neither under basal conditions nor following stimulation with LPS or TNF-α (Figure 4A, Table S4). Similarly, neither AN nor FV significantly altered TNF-α-induced NF-κB activation in HEK-Blue hTLR4 cells (Figure 4B).

In contrast, both AN and FV attenuated LPS-induced NF-κB activation in HEK-Blue hTLR4 cells (Figure 4B). Specifically, AN reduced NF-κB activity to 0.72-fold of the LPS control (*p* = 0.0009), while FV reduced NF-κB activation to 0.74-fold of the LPS control (*p* = 0.0022). This inhibitory effect was selective for LPS-mediated stimulation and was not observed under TNF-α challenge, indicating that AN and FV primarily inhibit TLR4-dependent NF-κB signaling rather than downstream, TLR4-independent NF-κB activation pathways.

### 2.5. AN and FV Reduce Intestinal Barrier Damage and Mortality in D. melanogaster

To validate the protective effects observed in vitro, AN and FV were evaluated in vivo using a dextran sulfate sodium (DSS)-induced intestinal barrier disruption model in female *D. melanogaster*.

**Figure 5:**
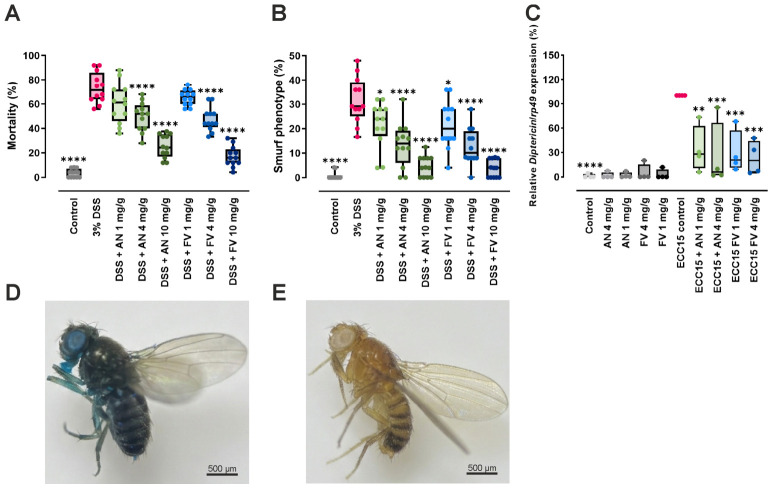
AN and FV protect intestinal barrier integrity and suppress Ecc15-induced *Diptericin* expression in female *D. melanogaster*. (**A**) Mortality (%) and (**B**) Smurf phenotype (%) observed in *D. melanogaster* fed with blue dye, challenged with 3% DSS and treated with AN and FV for 7 days. Statistical analysis was performed using one-way ANOVA with Tukey’s multiple comparison test. (**C**) Relative *Diptericin* expression (% of Ecc15-infected control) in flies following oral infection with Ecc15 and co-treatment with AN or FV extracts. Statistical analysis was performed using one-way ANOVA with Šídák’s multiple comparisons test. (**D**,**E**) Representative images of flies with disrupted (**D**, Smurf) and intact (**E**, non-Smurf) intestinal barriers. Data are shown as median with interquartile range (IQR), whiskers (min/max values), and individual data points from three independent experiments (**A**,**B**: *n* = 300 flies/treatment), and four independent experiments (**C**: *n* = 40 flies/treatment). Statistical significance is indicated as * *p* ≤ 0.05, ** *p* ≤ 0.01, *** *p* ≤ 0.001, and **** *p* ≤ 0.0001, with asterisks referring to comparisons against the DSS-stressed control group (**A**,**B**), and Ecc15-infected control group (**C**).

Intestinal barrier disruption was induced using 3% DSS, while AN or FV were co-treated as dietary supplements to evaluate their potential to preserve or restore barrier integrity. DSS exposure increased intestinal permeability, allowing a non-absorbable blue dye added to the diet to leak from the gut lumen into the hemocoel. This systemic dissemination of the dye resulted in the characteristic “Smurf” phenotype, which served as a visual readout of barrier failure (Figure 5D).

Mortality and intestinal barrier leakage (Smurf phenotype) were analyzed separately, as not all DSS-treated flies displayed visible dye leakage. DSS stress increased mortality compared to untreated controls (73.33% vs. 3.39%, *p* < 0.0001), confirming severe intestinal injury (Figure 5A). Co-treatment with AN or FV significantly improved survival at 4 and 10 mg/g in a dose-dependent manner. AN reduced mortality to 49.85% at 4 mg/g and 25.43% at 10 mg/g (both *p* < 0.0001 vs. DSS), whereas FV decreased mortality to 47.44% at 4 mg/g and 17.11% at 10 mg/g (both *p* < 0.0001 vs. DSS). Notably, FV at 10 mg/g reduced mortality to approximately one-quarter of that observed in DSS-treated flies. In contrast, 1 mg/g of either extract did not significantly affect mortality.

Intestinal barrier integrity was quantified by assessing the proportion of Smurf phenotype flies (Figure 5B). DSS significantly increased the proportion of flies exhibiting intestinal leakage compared to controls (31.58% vs. 0.68%, *p* < 0.0001). Both AN and FV significantly reduced the Smurf phenotype at all tested concentrations. At 10 mg/g, AN and FV decreased barrier leakage to 4.72% and 3.71%, respectively (both *p* < 0.0001 vs. DSS), approaching control levels. At 4 mg/g, leakage was reduced to 13.39% (AN) and 12.69% (FV) (both *p* < 0.0001), while 1 mg/g resulted in a modest but significant reduction (AN: 21.68%, *p* = 0.0356; FV: 21.67%, *p* = 0.0351).

Overall, both AN and FV significantly attenuated DSS-induced intestinal barrier disruption, with the most pronounced protective effects observed at 10 mg/g. FV exerted the strongest effect on survival, whereas both extracts comparably improved intestinal barrier integrity at higher concentrations.

### 2.6. AN and FV Reduce Ecc15-Induced Diptericin Expression in D. melanogaster

To investigate whether the protective effects of AN and FV extend to modulation of intestinal immune responses, we assessed their impact on intestinal immune signaling in *D. melanogaster*. Intestinal inflammation was induced by oral infection with *Erwinia carotovora carotovora* 15 (Ecc15), which activates the Imd/NF-κB signaling pathway. Expression of the antimicrobial peptide gene *Diptericin*, a canonical downstream target of this pathway, was quantified by qPCR and normalized to the housekeeping gene *rp49 (*Figure 5C*)*.

Ecc15 infection significantly induced *Diptericin* expression (set to 100%), confirming activation of the Imd/NF-κB pathway (*p* < 0.0001 vs. uninfected control). Co-treatment with AN or FV significantly attenuated *Diptericin* induction in a dose-dependent manner. At 4 mg/g, AN reduced *Diptericin* expression to 24.95% (*p* = 0.0003), and FV to 23.36% (*p* = 0.0002) relative to Ecc15 alone. Even at the lower 1 mg/g dose, AN and FV significantly decreased *Diptericin* expression to 33.86% (*p* = 0.0016) and 29.82% (*p* = 0.0007), respectively.

In uninfected flies, AN and FV did not significantly alter basal *Diptericin* expression, indicating that the extracts do not trigger immune activation in the absence of infection.

To ensure that the observed effects were due to regulation of host immune signaling rather than direct antibacterial activity, the ability of AN and FV to inhibit bacterial growth was tested using the Clinical and Laboratory Standards Institute (CLSI) broth microdilution method [28]. No inhibitory effect on Ecc15 growth was detected (Figure S1), confirming that the reduction in *Diptericin* expression reflects true immunomodulatory activity of the brown algae extracts.

These results demonstrate that AN and FV attenuate Ecc15-induced activation of the Imd/NF-κB pathway, a key pro-inflammatory signaling cascade in *D. melanogaster*, with the strongest suppression observed at 4 mg/g, reducing *Diptericin* expression to approximately one-quarter of the infected control. These findings complement the previously observed protective effects on intestinal barrier integrity and survival under DSS-induced stress, suggesting that both brown algae extracts modulate intestinal immune signaling while supporting gut barrier function in *D. melanogaster*.

## 3. Discussion

In this study, we investigated the anti-inflammatory and barrier-protective properties of aqueous extracts from the brown macroalgae *A. nodosum* (AN) and *F. vesiculosus (*FV*).* Using complementary in vitro macrophage and reporter cell models together with the in vivo *D. melanogaster* system, we demonstrate that both extracts attenuate LPS-induced inflammatory signaling, selectively inhibit TLR4-dependent NF-κB activation, reduce pro-inflammatory cytokine and chemokine release, and preserve intestinal barrier integrity under chemical and bacterial challenge.

These findings build directly on our previous study, in which the same aqueous extracts, AN and FV, were chemically characterized and shown to exert antioxidant activity in vitro and in vivo [25]. FV exhibited higher total polyphenolic content and antioxidant capacity, while AN contained higher levels of polysaccharides. LC-HRMS/MS analysis indicated greater diversity and polymerization of phlorotannins in FV extract. Because the extracts in the present study are identical to those previously characterized, the observed anti-inflammatory effects can be interpreted in the context of their defined chemical composition. The interplay between oxidative stress and inflammation is well established, as ROS generated during immune activation can amplify NF-κB-dependent pro-inflammatory gene expression [29]. The antioxidant activity previously observed likely contributes mechanistically to the attenuation of inflammatory signaling demonstrated here, suggesting that the antioxidant and anti-inflammatory effects of AN and FV represent complementary facets of a coordinated bioactivity profile.

Both AN and FV significantly reduced NO production in LPS-stimulated RAW264.7 macrophages in a concentration-dependent manner, with FV generally exhibiting stronger inhibition at higher doses. NO, produced via iNOS in response to TLR4/NF-κB activation, is a key pro-inflammatory mediator whose excess can amplify inflammation through reactive nitrogen species [30]. In the present study, both AN and FV significantly reduced LPS-induced NO production in a concentration-dependent manner, with *F. vesiculosus* generally exerting a stronger inhibitory effect at higher concentrations. Our findings align with previous reports that phlorotannin-rich fractions from *F. vesiculosus* and sulfated polysaccharides from *A. nodosum* independently suppress NO production and iNOS expression in macrophages [19]. Considering that the aqueous extracts used in the present study contain complex mixtures of polyphenolic compounds and polysaccharides, the observed NO-lowering effects likely reflect complementary, potentially synergistic regulation of inflammatory pathways, with phlorotannins acting predominantly via antioxidant effects and NF-κB inhibition, and polysaccharides interfering with upstream TLR4-mediated signaling.

To further characterize the immunomodulatory properties of AN and FV, we analyzed cytokine and chemokine secretion profiles in LPS-stimulated THP-1 macrophages using a semi-quantitative cytokine array. LPS stimulation markedly increased the release of numerous inflammatory mediators, particularly those linked to NF-κB-dependent signaling and prostaglandin E_2_/I_2_ pathways, consistent with previous observations [31]. Treatment with AN or FV altered the LPS-induced secretory profile, leading to an overall reduction in multiple pro-inflammatory markers, with FV generally exerting a stronger suppressive effect than AN, indicating higher efficacy in limiting LPS-driven inflammatory signaling. These differences were primarily quantitative rather than qualitative, as both extracts affected largely overlapping mediator profiles.

To quantitatively validate the array-based observations, selected cytokines and chemokines were further analyzed using a bead-based multiplex immunoassay, providing a more sensitive and precise assessment of key inflammatory mediators. Multiplex bead-based analysis validated reductions in TNF-α, IL-6, IL-33, CXCL9, CXCL10, CXCL11, and CCL7, revealing dose-dependent and mediator-specific differences between the extracts. Notably, AN sometimes showed stronger inhibition at lower doses for certain chemokines, suggesting that polysaccharides may contribute to early or low-concentration effects, while phlorotannin-rich FV provides more pronounced suppression at higher doses.

Several studies have documented the anti-inflammatory potential of brown algae, including *A. nodosum* and *F. vesiculosus*, though most focused on classical mediators such as TNF-α, IL-6, and IL-1β [32,33]. Fucoidan extracts from these species decrease LPS-induced TNF-α and IL-6 secretion in immune cells, supporting suppression of NF-κB and MAPK pathways [34], while purified *F. vesiculosus* fucoidan also enhanced anti-inflammatory IL-10 in vitro and in vivo [35]. Brown algae extracts have also been shown to modulate other inflammatory mediators; for example, *A. nodosum* and *F. vesiculosus* polysaccharide fractions have demonstrated inhibitory effects on LPS-induced PGE2 and COX-2 expression as well as downstream inflammatory signaling in ex vivo and cell culture models [36].

Although previous studies focused on a narrow panel of markers, our findings show that AN and FV exert broader anti-inflammatory effects across a wider set of cytokines and chemokines than typically investigated. In addition to classical pro-inflammatory cytokines both extracts significantly reduced CXCL9, CXCL10, CXCL11, CCL7, and the alarmin IL-33 in LPS-stimulated THP-1 macrophages. CXCR3-axis chemokines (CXCL9, CXCL10, CXCL11) regulate T and NK cell recruitment, CCL7 directs monocyte and lymphocyte trafficking, and IL-33 amplifies innate and type 2 responses in chronic inflammation [37,38]. Supporting evidence from other botanical models indicates that attenuation of chemokine and alarmin signaling can have meaningful effects on disease outcomes. For example, ginger extract reduced IL-33 expression, as well as IL-27, in the central nervous system and ameliorated clinical severity in a mouse model of experimental autoimmune encephalomyelitis, suggesting that suppression of IL-33 may contribute to reduced inflammatory pathology in complex immune environments [39]. Similarly, Moutan Cortex Radicis (MCR) extract was reported to down-regulate CXCL9, CXCL10, and CXCL11 gene expression in LPS-stimulated gingival fibroblasts, demonstrating that plant-derived extracts can modulate CXCR3 ligands and other chemokines in addition to classical pro-inflammatory cytokines [40].

To explore the potential signaling pathways contributing to the anti-inflammatory activity of AN and FV, we assessed NF-κB activation in HEK-Blue hTLR4 reporter cells, which express human TLR4, and in TLR4-deficient HEK-Blue null2 cells. Both extracts selectively attenuated LPS-induced NF-κB activation in hTLR4 cells without affecting TNF-α signaling or NF-κB activity in null2 cells, indicating pathway-specific inhibition of TLR4-dependent signaling. Such pathway-selective modulation may help to maintain basal immune functions while limiting excessive inflammatory responses. Similar TLR4-targeted effects have been reported for other brown algae compounds, including *Ecklonia cava* extracts and fucoidans, which suppress NO production and NF-κB activation via TLR/MyD88 pathways [41]. Although these studies used different assays, they align with our findings by demonstrating that brown algae bioactives target early steps of TLR4-mediated signaling rather than general NF-κB suppression.

The selective attenuation of TLR4-dependent NF-κB activation by AN and FV in HEK-Blue reporter cells indicates that part of their anti-inflammatory activity arises from interference with LPS-triggered TLR4 signaling. However, NF-κB inhibition alone likely does not fully explain the broad suppression of chemokines and cytokines observed in THP-1 macrophages. Bioactive constituents of brown algae, such as fucoidans and phlorotannins, can attenuate multiple inflammatory pathways beyond NF-κB. For example, fucoidan fractions suppress NF-κB and MAPK signaling in LPS-activated macrophages, reducing pro-inflammatory mediator expression via downregulation of p38, ERK, and JNK cascades as well as NF-κB nuclear translocation [42]. Phlorotannins similarly inhibit MAPK and Akt pathways, attenuating inflammatory mediator production and affecting transcription factors and kinases beyond NF-κB [43]. Other brown algal bioactives, such as fucoxanthin, activate Nrf2/HO-1 antioxidant pathways while concurrently inhibiting NF-κB, indicating crosstalk between oxidative stress and inflammatory signaling networks [44]. Thus, the aqueous extracts AN and FV, which contain a complex mixture of polysaccharides, polyphenols, and other minor bioactives, likely reduce chemokine and cytokine secretion not only via TLR4-NF-κB blockade but also by engaging MAPKs, Nrf2/HO-1, and additional regulatory pathways. This multi-target modulation may explain the extensive inhibition of chemokines such as CXCL9, CXCL10, CXCL11, and CCL7, which are controlled by signaling networks beyond canonical NF-κB.

The *D. melanogaster* experiments provide in vivo confirmation that the immunomodulatory properties of AN and FV observed in mammalian macrophages translate to a whole-organism context. Oral infection with Ecc15 robustly induced *Diptericin*, a canonical Imd/NF-κB target, confirming innate immune activation. Both extracts significantly and dose-dependently reduced *Diptericin* expression, while basal levels in uninfected flies remained unaffected. This indicates that AN and FV do not constitutively stimulate immune pathways but rather suppress excessive immune activation under challenge conditions. The Imd pathway in *D. melanogaster* is functionally analogous to mammalian TNFR- and TLR-dependent NF-κB signaling: recognition of Gram-negative bacteria triggers NF-κB transcription factor Relish activation, inducing antimicrobial peptides like *Diptericin* [45]. The strong reduction in *Diptericin* expression to approximately 25–30% of infected controls suggests interference with NF-κB-mediated transcription or upstream receptor-dependent signaling, consistent with our HEK-Blue reporter data and supporting the idea that these extracts target evolutionarily conserved innate immune pathways. Importantly, *Diptericin* expression was attenuated rather than completely abolished, indicating selective dampening of the immune response rather than general immune suppression.

The DSS-induced Smurf assay further demonstrated that these molecular effects translate into functional protection of gut integrity. DSS exposure caused severe epithelial barrier disruption, increased intestinal permeability, and elevated mortality. Co-treatment with AN and FV significantly reduced the proportion of Smurf-positive flies and improved survival in a dose-dependent manner, with the strongest protection observed at 10 mg/g. FV exerted the most pronounced effect on survival, whereas both extracts comparably restored barrier integrity at higher concentrations.

Notably, the concentrations of AN and FV used in the *D. melanogaster* experiments were higher than those applied in vitro, reflecting the increased physiological complexity of the whole organism, including metabolic processing and potential microbiota interactions. In addition, female flies were used in the Smurf assays for technical reasons, primarily to facilitate reliable visual identification of the Smurf phenotype, rather than to assess sex-specific differences in response to the extracts.

In *D. melanogaster*, DSS exposure compromises epithelial integrity and triggers oxidative stress alongside activation of innate immune pathways, including NF-κB/Relish-dependent responses, contributing to intestinal barrier dysfunction and inflammation [46]. The combined reduction in *Diptericin* expression and barrier leakage indicates that AN and FV interrupt this cycle, limiting inflammation-driven tissue damage while stabilizing epithelial integrity. Given that aqueous brown algae extracts contain polysaccharides such as fucoidans and polyphenolic compounds, this protection likely results from coordinated anti-inflammatory and cytoprotective mechanisms, potentially involving modulation of oxidative stress and epithelial resilience in addition to NF-κB regulation.

To our knowledge, this study is the first to investigate the effects of brown algae extracts on intestinal immune signaling and barrier integrity in *D. melanogaster*. While previous research on brown algae in the fly has primarily focused on lifespan extension or metabolic outcomes following supplementation [47,48], our approach combines both chemical (DSS) and bacterial (Ecc15) challenges to model intestinal stress and immune activation. By assessing functional endpoints such as Smurf phenotype-associated barrier leakage and NF-κB-dependent *Diptericin* expression, we provide direct evidence that AN and FV restrain inflammatory signaling while preserving gut barrier integrity. The parallels between fly Imd/NF-κB pathways and mammalian TLR/NF-κB signaling, together with the conserved roles of oxidative stress and inflammatory mediators in epithelial damage, suggest these mechanisms may be evolutionarily conserved and relevant to higher organisms.

In addition to their immunomodulatory and barrier-protective effects, brown algae polysaccharides and polyphenols may influence intestinal homeostasis by altering gut microbial communities. Fucoidans and phlorotannins have been reported to promote the growth of beneficial commensals such as *Lactobacillus* and *Bifidobacterium*, potentially contributing to reduced inflammation and improved epithelial integrity [49,50]. In *D. melanogaster*, the gut microbiota is relatively simple but plays a key role in regulating immunity and gut function [51]. Although microbial composition was not assessed in this study, future work could clarify whether microbiota-mediated effects contribute to the protective properties of AN and FV.

Another limitation is that, although *D. melanogaster* offers a convenient and evolutionarily conserved model to study innate immunity and epithelial barrier function, it lacks adaptive immunity, and caution is warranted when extrapolating findings to mammalian systems [52]. Additionally, our experiments focused on short-term supplementation and acute challenges, leaving long-term effects on growth, metabolism, immunity, or microbiota unexplored. The extracts represent complex mixtures of bioactive compounds, including polysaccharides, polyphenols, and minor constituents, and the relative contributions of individual components were not dissected. Finally, while we observed clear anti-inflammatory and barrier-protective effects, the specific molecular targets and pathways beyond NF-κB, such as MAPKs, and Nrf2/HO-1 signaling warrant further mechanistic exploration. Addressing these limitations in future work will strengthen understanding of the mechanisms underlying the beneficial effects of brown algae extracts and their translational relevance as dietary interventions for gut health.

Although brown algae are already used as feed ingredients in animal nutrition, the molecular mechanisms underlying their beneficial effects on intestinal health remain incompletely understood. In addition, considerable variability exists among algae-based products with respect to species composition, harvest conditions, processing procedures, extraction methods, and bioactive compound content, making direct comparisons between studies and the establishment of optimal supplementation strategies challenging. While precise dose extrapolation to livestock species such as pigs or broilers remains difficult, previous feeding studies with *Ascophyllum nodosum* have nevertheless reported beneficial effects on gut health, immune function, oxidative status, and physiological performance at dietary inclusion levels ranging from approximately 0.5 to 10 g/kg feed, depending on the preparation and experimental endpoint assessed [53,54,55,56,57]. These differences likely reflect variation in the concentration and composition of bioactive constituents, including phlorotannins, fucoidans, and other polysaccharides. Consequently, optimal supplementation levels are likely highly product-specific.

The present findings provide mechanistic evidence that AN and FV restrict NF-κB-dependent inflammatory signaling and support epithelial barrier integrity. Further studies in mammalian models and livestock species will be important to refine dosage strategies, evaluate long-term physiological effects, and determine how these bioactive compounds influence gut health under practical production conditions.

## 4. Materials and Methods

### 4.1. Sample Preparation

Dried *A. nodosum* powder with a mean particle size of 100 µm and *F. vesiculosus* meal with a particle size of 1–2 mm were supplied by NUQO© (Annecy, France). The *A. nodosum* material was provided in a pre-micronized form and was therefore used without additional size reduction. In contrast, the *F. vesiculosus* meal was milled using a laboratory coffee grinder to obtain a particle size comparable to that of *A. nodosum* prior to extraction. Extracts from both brown algal materials were prepared by solid–liquid extraction as previously described [25]. In brief, the algal powders were suspended in deionized water at a mass ratio of 1:10 (*w*/*w*). Deionized water was chosen as the extraction medium because of its safety, lack of toxicity, and suitability for feed-related applications, where the presence of organic solvent residues is not acceptable. The suspensions were maintained at ambient temperature under continuous overhead stirring for 24 h. After extraction, the mixtures were centrifuged at 16,000× *g* for 30 min, and the resulting supernatants were recovered. The dry matter content of the extracts was determined using an infrared moisture analyzer (Sartorius, Göttingen, Germany). All liquid extracts were stored at −20 °C until analysis. The aqueous extracts from *A. nodosum* and *F. vesiculosus* are referred to as AN and FV, respectively. Algae extract concentrations are expressed relative to the amount of initial macroalgal material per volume of water.

### 4.2. Cell Culture Maintenance

The murine macrophage-like cell line RAW264.7 (ATCC, Manassas, VA, USA) and the human monocytic cell line THP-1 (DSMZ, Braunschweig, Germany) were maintained at 37 °C in a humidified atmosphere containing 5% CO_2_. RAW264.7 cells were cultured in Dulbecco’s Modified Eagle Medium (DMEM; 4.5 g/L glucose, stable glutamine, sodium pyruvate, and 3.7 g/L sodium bicarbonate), whereas THP-1 cells were grown in Roswell Park Memorial Institute medium (RPMI 1640) supplemented with 2 mM L-glutamine, 1 mM sodium pyruvate, 10 mM HEPES, 4.5 g/L glucose, 1.5 g/L sodium bicarbonate, and 0.05 µM 2-mercaptoethanol. Both media were supplemented with 10% fetal bovine serum (FBS) and penicillin–streptomycin (100 U/mL and 100 µg/mL, respectively; PAN-Biotech, Aidenbach, Germany). Cells were passaged twice weekly. For differentiation into macrophage-like cells, THP-1 monocytes were treated with phorbol 12-myristate 13-acetate (PMA; 50 ng/mL; Sigma-Aldrich, St. Louis, MO, USA) for 48 h, resulting in the transition from a suspension culture to an adherent phenotype. Differentiation efficiency was assessed based on cell adherence, with approximately 90% adherent cells considered indicative of successful maturation. All experiments involving THP-1 cells were conducted using differentiated cells.

The HEK-Blue hTLR4 reporter cell line, which constitutively expresses the human toll-like receptor 4 (TLR4) together with an NF-κB-responsive secreted embryonic alkaline phosphatase (SEAP) reporter, and its corresponding control cell line HEK-Blue null2 (expressing SEAP only) were obtained from InvivoGen (San Diego, CA, USA). These cells were cultured in DMEM containing 4.5 g/L glucose, stable glutamine, sodium pyruvate, and 3.7 g/L sodium bicarbonate under the same incubation conditions described above. The culture medium was supplemented with 10% heat-inactivated FBS, penicillin–streptomycin (100 U/mL-100 µg/mL), 100 µg/mL Normocin™ (InvivoGen), and the appropriate selection antibiotics: 1× HEK-Blue Selection for HEK-Blue hTLR4 cells or 100 µg/mL Zeocin for HEK-Blue null2 cells.

All cell culture experiments were carried out using THP-1 and RAW264.7 cells up to passage 35 and HEK-Blue reporter cells up to passage 20.

### 4.3. NO Quantification in RAW264.7 Macrophages

NO release by RAW264.7 macrophages was evaluated following stimulation with LPS in the presence or absence of the test compounds. The assay is based on the quantification of nitrite (NO_2_^−^), a stable oxidation product of NO, in cell culture supernatants. Under acidic conditions, nitrite reacts with sulfanilamide and N-(1-naphthyl)ethylenediamine dihydrochloride (NED) to form a chromophoric azo dye, the intensity of which is proportional to the nitrite concentration [58]. NO measurements were performed using the Griess Reagent System (Promega Corporation, Fitchburg, WI, USA) according to the manufacturer’s instructions, with slight modifications as previously reported [59]. In brief, RAW264.7 cells were plated in 96-well plates (Greiner Bio-One GmbH, Kremsmünster, Austria) at a density of 6.4 × 10^4^ cells per well (triplicates) and allowed to adhere overnight at 37 °C. Cells were subsequently stimulated with LPS (250 ng/mL; Sigma-Aldrich) to induce NO production, either alone or in combination with AN or FV at concentrations of 10, 50, 100, 200 and 400 µg/g. Quercetin (20 µM) was included as a positive control. Following a 24 h incubation at 37 °C in a humidified atmosphere containing 5% CO_2_, equal volumes (50 µL) of culture supernatant or nitrite standards were mixed with 50 µL of sulfanilamide reagent and incubated for 10 min at room temperature. Subsequently, 50 µL of NED solution was added and the reaction was allowed to proceed for an additional 10 min. Absorbance was measured at 548 nm using a POLARstar Omega microplate reader (BMG Labtech, Ortenberg, Germany). Nitrite concentrations were calculated from the standard curve, background-subtracted, and expressed relative to the LPS-treated control.

### 4.4. Analysis of Inflammatory Cytokine Secretion by LPS-Challenged THP-1 Cells

#### 4.4.1. Sample Collection Following LPS Stimulation of THP-1 Cells

THP-1 monocytes were plated in duplicate in 6-well culture plates at a density of 2.7 × 10^6^ cells per well and differentiated into macrophage-like cells by exposure to 50 ng/mL PMA for 48 h. Test compounds were prepared in serum-free culture medium to obtain a final concentration of 100 and 400 µg/g, and 250 ng/mL LPS was included to induce an inflammatory response. Prior to treatment, cells were rinsed with phosphate-buffered saline (PBS; PAN-Biotech) and subsequently incubated with 2.5 mL of the respective treatment solutions for 24 h. Following incubation, culture supernatants were harvested and clarified by centrifugation at 200× *g* for 4 min. The cleared supernatants were stored at −80 °C until further analysis of cytokine release.

#### 4.4.2. Cytokine Array Profiling

The expression patterns of cytokines, chemokines, and growth factors were examined using the semi-quantitative Human XL Cytokine Array (Proteome Profiler^TM^; Bio-Techne Ltd., Abingdon, UK), according to the manufacturer’s instructions. In brief, array membranes were blocked for 60 min at room temperature and subsequently incubated with the samples overnight at 4 °C. After extensive washing, the membranes were exposed to biotin-labeled detection antibodies for 90 min at room temperature, followed by incubation with streptavidin–horseradish peroxidase for 30 min under the same conditions. Protein signals were visualized using chemiluminescent substrates applied for 1 min, and emitted light was captured with a ChemiDoc MP Imaging System (Bio-Rad Laboratories, Hercules, CA, USA). Signal intensities were quantified using the Image Lab software 6.1 (Bio-Rad Laboratories). Pixel intensity values for each array spot were exported and processed in R (R Foundation for Statistical Computing, Vienna, Austria), where a global background correction was applied. The standard deviation (SD) was calculated for each analyte and treatment. Signals affected by spillover from adjacent high-intensity spots were removed from the dataset. Analytes with mean signal intensities below the limit of detection (LOD), defined as the global background plus three times its SD, were excluded from further evaluation and classified as not detected. Log_2_-transformed fold changes between treatment groups were calculated and imported into Cytoscape (version 3.9.1) (Institute for Systems Biology) for downstream visualization and network analysis.

#### 4.4.3. Cytokine Multiplex Immunoassay

Levels of selected pro-inflammatory cytokines and chemokines including tumor necrosis factor α (TNF-α), interleukin (IL)-6, IL-33, CC chemokine ligand 7 (CCL7), C-X-C motif chemokine ligands CXCL9, CXCL10, and CXCL11 released into the culture medium of LPS-stimulated THP-1 cells were measured using a customized 7-plex Luminex xMAP^®^ assay (Luminex Corp., Austin, TX, USA), conducted in accordance with the manufacturer’s instructions. Culture supernatants were analyzed undiluted as well as after dilution at ratios of 1:5 and 1:20 to ensure measurements within the dynamic range of the assay. For the assay procedure, 50 µL of standards or samples were incubated with an equal volume of MagPlex™ microspheres pre-coupled with capture antibodies (Luminex Corp.) for 2 h. The beads were subsequently washed and incubated with 50 µL of biotin-conjugated detection antibodies for 1 h. After another wash step, streptavidin–phycoerythrin was added and allowed to bind for 30 min. The beads were then washed again and resuspended in 100 µL of wash buffer prior to analysis. All incubation steps were carried out in sealed, light-protected, black-bottom microplates on a plate shaker operating at 810 rpm. Data acquisition was performed using a Luminex^®^ 200™ analyzer (Luminex Corp.), and results were processed using the xPONENT^®^ acquisition software (version 4.3; Luminex Corp.).

### 4.5. Assessment of NF-κB Signaling in HEK-Blue Reporter Cells

Activation of NF-κB in response to LPS, tumor necrosis factor α (TNF-α), and the brown algae extracts was evaluated using the HEK-Blue hTLR4 reporter cell line together with its corresponding parental control cell line, HEK-Blue null2. NF-κB activity was quantified by measuring the levels of SEAP, a downstream reporter of NF-κB activation. The inclusion of the null2 cell line enabled discrimination between TLR4-dependent and -independent signaling events. For the assay, 20 µL of LPS, TNF-α, and/or AN or FV (prepared in DMEM supplemented with 10% heat-inactivated FBS, 100 U/mL-100 µg/mL penicillin-streptomycin, and 100 µg/mL Normocin™) were dispensed into 96-well plates. Final concentrations were 10 ng/mL for LPS or TNF-α and 200 µg/g AN or FV. Subsequently, 180 µL of HEK-Blue hTLR4 cells or HEK-Blue null2 cells were added in triplicate at densities of 1 × 10^5^ and 0.6 × 10^5^ cells per well, respectively. After 24 h of incubation at 37 °C, 20 µL of culture supernatant from each well were transferred to 180 µL of QUANTI-Blue reagent (InvivoGen), which contains the chromogenic substrate for SEAP. The reaction mixtures were incubated for 30 min at 37 °C, and absorbance was recorded at 620 nm using a POLARstar Omega microplate reader. Cell viability was assessed in parallel using the CellTiter-Glo^®^ Cell Viability Assay (Promega Corporation) according to the manufacturer’s protocol. Data were background-subtracted and normalized to untreated control.

### 4.6. D. melanogaster for Studying Protective Effect of AN and FV

#### 4.6.1. *D. melanogaster* Husbandry and Strains

For intestinal barrier challenge assays, *D. melanogaster* wild-type flies (w^1118^; Bloomington Drosophila Stock Center, Bloomington, IN, USA; stock no. 5905), originally obtained from the University of Kiel (Kiel, Germany), were maintained under controlled laboratory conditions (25 °C, 60% relative humidity, and a 12 h light/dark cycle). Flies were reared on larval growth medium and sugar–yeast medium as previously described [60]. Unless stated otherwise, all reagents used for intestinal barrier challenge experiments were sourced from Carl Roth GmbH.

For the infection assay, adult *w;Gla/CyO* flies (Bloomington Drosophila Stock Center; stock no. 5439) were used, which were maintained at 25 °C on medium containing agar 0.6% (*w*/*v*), malt 6.5% (*w*/*v*), semolina 3.2% (*w*/*v*), baker’s yeast 1.8% (*w*/*v*), nipagin 2.4%, and propionic acid 0.7% (Hi-Fly University of Helsinki Drosophila core facility, Helsinki Institute of Life Science) in a 12 h light/dark cycle.

#### 4.6.2. Intestinal Barrier Challenge (Smurf Assay)

The protective effects of AN and FV on intestinal barrier integrity were evaluated in vivo using a DSS-induced gut permeability (“Smurf”) assay in *D. melanogaster*. The experimental design was adapted from established protocols [61] with modifications from our previous work [62].

Female w^1118^ flies, 5 days old, were sorted into experimental groups of 25 ± 2 flies per vial. Prior to treatment, flies were transferred to vials containing 2% agar to maintain humidity and prevent desiccation of the liquid feeding medium. Experimental diets were provided via 1.5 mm thick blotting paper (Whatman, Cytiva, UK) mounted on cellulose acetate plugs (Genesee Scientific, Morrisville, UK) and soaked with liquid media.

The liquid experimental medium consisted of 5% sucrose, 0.5% Brilliant Blue FCF (C.I. 42090) and 3% DSS (average molecular weight 40,000 g·mol^−1^) in tap water. Treatment groups additionally received either AN or FV at concentrations of 1 mg/g, 4 mg/g, or 10 mg/g. Control groups were supplied with sucrose and Brilliant Blue FCF without DSS, while DSS-only groups served as challenge controls.

Four vials (~100 flies) per treatment were used per experiment. Three independent experiments were conducted, resulting in a total of approximately 300 flies per treatment across all replicates. Flies were maintained under standard laboratory conditions (25 °C, 60% relative humidity) for 7 days. Feeding substrates and agar vials were replaced four times during the experimental period to ensure consistent exposure to fresh media.

Fly survival and intestinal barrier integrity were recorded daily, beginning on day 3 of the assay. The Smurf phenotype was defined as the presence of blue dye outside the intestinal tract, such as in the thorax, head, or appendages, indicating loss of gut barrier function.

#### 4.6.3. Bacterial Strain and Infection Assay

The Gram-negative bacterium Ecc15 was generously provided by Dr. François Leulier. For septic infection, Ecc15 was cultured in LB medium at 29 °C with continuous shaking for 16 h and adjusted to an optical density (OD_600_) of 0.2. Oral infection in adult flies was induced by feeding a 1:1 solution of Ecc15 and 5% sucrose in the presence and absence of 1 mg/g or 4 mg/g of AN or FV. For subsequent quantitative PCR (qPCR) analysis, 10 adult flies per condition were incubated for 5 h at 25 °C following infection.

#### 4.6.4. Quantitative RT-PCR (qPCR)

Ten adult flies were homogenized using QIAshredder (Qiagen, Hilden, Germany) and total RNA was extracted with RNeasy Mini Kit (Qiagen) and cDNA was synthesized with a SensiFast cDNA synthesis kit (Bioline, London, UK) according to the manufacturers’ protocols. qPCR was performed using SensiFast SYBR Hi-ROX qPCR kit (Bioline). *rp49* was used as a housekeeping gene for ∆∆Ct calculations. The following gene-specific primers were used to amplify cDNA: *Diptericin* (5′-ACCGCAGTACCCACTCAATC, 5′-ACTTTCCAGCTCGGTTCTGA), *rp49* (5′-GACGCTTCAAGGGACAGTATCTG, 5′-AAACGCGGTTCTGCATGAG). The results from qPCR were normalized to the Ecc15 stress control sample.

### 4.7. Statistical Analysis

Statistical analyses were performed using Microsoft Excel 365 (Microsoft Corporation, Redmond, WA, USA) and GraphPad Prism version 10.2.2 (GraphPad Software, Boston, MA, USA). Prior to hypothesis testing, datasets were screened for outliers using the ROUT method (Q = 0.5%). Data distribution was assessed using normality tests to determine whether values followed a Gaussian distribution. For comparisons involving more than two groups with equal variances, ordinary one-way analysis of variance (ANOVA) was applied, followed by Tukey’s multiple comparison test to control the family-wise error rate across all pairwise comparisons. If the assumption of homogeneity of variances was violated, Brown–Forsythe and Welch ANOVA were performed. In these cases, Dunnett’s multiple comparison test was used when experimental groups were compared against a single control group, as this approach is specifically designed to control type I error in multiple-to-control comparisons. For selected analyses involving predefined pairwise comparisons, Šídák’s multiple comparisons test was applied to adjust for multiple testing while maintaining statistical power. Unless otherwise stated, asterisks indicate comparisons between two groups. Significant *p* values are indicated as * (≤ 0.05), ** (≤ 0.01), *** (≤ 0.001), **** (≤ 0.0001) or ns (not significant).

## 5. Conclusions

This study shows that aqueous extracts of the brown algae *A. nodosum* and *F. vesiculosus* have immunomodulatory and gut barrier-protective effects in both in vitro and in vivo models. In macrophages, both extracts reduced LPS-induced nitric oxide production and the release of multiple inflammatory cytokines and chemokines. These effects are linked to selective modulation of TLR4-dependent NF-κB activation. In vivo, both extracts attenuated Ecc15-induced intestinal immune signaling and preserved epithelial barrier integrity in *D. melanogaster*. These effects are likely driven by polyphenols (e.g., phlorotannins) and polysaccharides (e.g., fucoidans), which modulate oxidative stress and inflammatory signaling pathways. While *F. vesiculosus* showed stronger effects at higher doses, *A. nodosum* was effective at lower concentrations. Overall, the findings highlight the potential of brown algae to support gut health.

## Data Availability

Data are available upon reasonable request.

## Supplementary Materials

The following supporting information can be downloaded at: https://www.mdpi.com/article/10.3390/md24050182/s1, Table S1: Total phenolic content (TPC), total polysaccharide content (TPSC), and antioxidant capacity (TEAC and FRAP) of AN and FV; Table S2: Tentatively identified phlorotannins (PTs) in AN; Table S3: Tentatively identified phlorotannins (PTs) in FV; Table S4: AN and FV do not affect NF-κB activation in HEK-Blue null2 cells under basal conditions or following stimulation with LPS; Figure S1: AN and FV do not alter bacterial growth at the tested concentrations. References [63,64,65,66,67,68] are cited in the supplementary materials.

## Abbreviations

The following abbreviations are used in this manuscript:

AGP	Antimicrobial growth promoters
AN	Aqueous extract of *Ascophyllum nodosum*
CCL	CC chemokine ligand
CXCL	C-X-C motif chemokine ligand
DSS	dextran sulfate sodium
Ecc	*Erwinia carotovora carotovora*
FBS	fetal bovine serum
FV	Aqueous extract of *Fucus vesiculosus*
Imd	immune deficiency
iNOS	inducible nitric oxide synthase
IL	interleukin
LOD	limit of detection
LPS	lipopolysaccharide
NED	N-(1-naphthyl)ethylenediamine dihydrochloride
NO	nitric oxide
NF-κB	nuclear factor kappa B
OD	optical density
PRRs	pattern recognition receptors
PMA	phorbol 12-myristate 13-acetate
PBS	phosphate-buffered saline
ROS	reactive oxygen species
SEAP	secreted embryonic alkaline phosphatase
SD	standard deviation
TLRs	toll-like receptors
TNF-α	tumor necrosis factor α
