# Supplementary material for: From Sea to Cell: Ascophyllum nodosum and Fucus vesiculosus Extracts Attenuate NF-κB-Mediated Inflammation and Protect Intestinal Barrier Integrity—A Comprehensive Analysis Applying In Vitro and In Vivo Models
Source: Mar Drugs. 2026 May 19;24(5):182. doi: 10.3390/md24050182 (PMC13208233; doi:10.3390/md24050182)
Supplement: Supplementary file 1 [file marinedrugs-24-00182-s001.zip › marinedrugs-4289233-supplementary.pdf]

marine drugs

Article

From Sea to Cell: *Ascophyllum nodosum* and *Fucus vesiculosus* Extracts Attenuate NF-κB–Mediated Inflammation and Protect Intestinal Barrier Integrity – a comprehensive analysis applying in vitro and in vivo models

Lea Karlsberger [1,2], Nadiia Sadova [1,2], Mara Heckmann [1,2], Fanny Serenius [3], Annika Meinander [3,4], Julia Kirchsteiger [2], Alice König [2], Bettina Schwarzinger [1,2], Bernhard Blank-Landeshammer [2], Stephanie Ladirat [5], and Julian Weghuber [1,2*]

[1] FFoQSI GmbH – Austrian Competence Centre for Feed and Food Quality, Safety and Innovation, Technopark 1D, 3430 Tulln, Austria
[2] Center of Excellence Food Technology and Nutrition, University of Applied Sciences Upper Austria, Stelzhamerstraße 23, Wels 4600, Austria
[3] Faculty of Science and Engineering, Biochemistry and Cell Biology, Åbo Akademi University, Henrikinkatu 2, 20500 Turku, Finland
[4] InFLAMES Research Flagship Center, Åbo Akademi University, Henrikinkatu 2, 20500 Turku, Finland
[5] SAS NUQO, Chez Axes Compta conseils, Immeuble l'ONYX, 28 Avenue du Pamelan, 74000-Annecy, France
* Correspondence: julian.weghuber@fh-wels.at

Academic Editors: Cinzia Parolini and Elda Favari

Received: 14 April 2026
Revised: 11 May 2026
Accepted: 14 May 2026
Published: 25 May 2026

Copyright: © 2026 by the authors. Licensee MDPI, Basel, Switzerland. This article is an open access article distributed under the terms and conditions of the Creative Commons Attribution (CC BY) license.

Supplementary Methods

Determination of total phenolic content (TPC)

Total phenolic content of aqueous extracts from *Ascophyllum nodosum* (AN) and *Fucus vesiculosus* (FV) was quantified using the Folin–Ciocalteu assay as previously reported [1]. Phloroglucinol was employed as the calibration standard. A stock solution (1 g/L) was serially diluted with distilled water to obtain calibration standards ranging from 10 to 200 mg/L.

For analysis, 100 μL of appropriately diluted extract (1:100), standard, or water (blank) was mixed with 1 mL distilled water, 100 μL Folin–Ciocalteu reagent, and 500 μL saturated sodium carbonate solution. Samples were vortexed and incubated in the dark at room temperature for 70 min. Subsequently, 200 μL of each reaction mixture was transferred to a 96-well microplate, and absorbance was recorded at 750 nm using a microplate reader. Measurements were performed in triplicate. Results were calculated from the calibration curve and expressed as mg phloroglucinol equivalents per g dry weight (mg PGE/g).

Determination of total polysaccharide content (TPSC)

Total polysaccharide content was determined using the phenol–sulfuric acid method as described previously [2]. D-glucose served as the reference compound for calibration (10–150 mg/L).

Briefly, 200 μL of diluted extract (1:100), standard, or water was combined with 600 μL concentrated sulfuric acid and 120 μL of 5% phenol solution in screw-cap tubes. After vortexing, samples were incubated at 85 °C for 20 min. Following incubation, 230 μL aliquots were transferred to a 96-well plate, and absorbance was measured at 490 nm. All samples were analyzed in triplicate. Polysaccharide content was calculated from the glucose calibration curve and expressed as mg glucose equivalents per g dry weight (mg GE/g).

Trolox equivalent antioxidant capacity (TEAC)

The antioxidant capacity of the extracts was evaluated using the ABTS radical cation decolorization assay following established protocols [3–5]. Trolox was used as the standard (25–750 μM).

ABTS radical cations ($ABTS^{\bullet +}$) were generated by reacting 7 mM ABTS with 2.45 mM potassium persulfate, followed by incubation in the dark for 16 h at room temperature. Prior to use, the solution was diluted with water to an absorbance of 0.70 ± 0.02 at 734 nm.

For measurement, 4 μL of diluted extract (1:10), standard, or blank was added to 196 μL of $ABTS^{\bullet +}$ solution in a 96-well plate. After 5 min incubation at room temperature, absorbance was recorded at 734 nm. All measurements were conducted in triplicate. Antioxidant capacity was expressed as μmol Trolox equivalents per g dry weight (μmol TE/g).

Ferric reducing antioxidant power (FRAP)

Ferric reducing antioxidant power was determined using a modified FRAP assay [6]. Trolox served as the calibration standard (25–750 μM).

The FRAP reagent was freshly prepared by mixing acetate buffer (300 mM, pH 3.6), ferric chloride solution (20 mM), and TPTZ solution (10 mM in 40 mM HCl) in a ratio of 10:1:1. The reagent was pre-warmed to 37 °C prior to use.

For analysis, 20 μL of sample, standard, or blank was combined with 180 μL of FRAP reagent in a 96-well plate. Absorbance was measured at 593 nm after 0, 4, and 10 min. All samples were analyzed in triplicate. Results were calculated using the Trolox calibration curve and expressed as μmol Trolox equivalents per g dry weight (μmol TE/g).

https://doi.org/10.3390/md24050182

LC-HRMS/MS analysis of phlorotannins

Phlorotannin profiles of AN and FV extracts were characterized by LC-HRMS/MS using both targeted and untargeted approaches. Freeze-dried extracts were dissolved in acetonitrile:water (10:90, v/v) to a concentration of 5 mg/mL, centrifuged, and the supernatants were transferred to LC-MS vials. Samples were prepared in triplicate and 1 µL was injected for analysis.

Chromatographic separation was performed on an Ascentis Express RP-Amide column (10 cm × 2.1 mm, 2 µm particle size) maintained at 30 °C, using an Agilent 1260 Infinity HPLC system. The mobile phases consisted of water with 0.2% formic acid (A) and acetonitrile with 0.2% formic acid (B), delivered at a flow rate of 0.25 mL/min. The gradient started at 10% B, increased to 100% B over 36 min, held for 14 min, and then re-equilibrated to initial conditions.

Mass spectrometric detection was performed using an LTQ Velos Pro Orbitrap Elite instrument equipped with an electrospray ionization source operating in negative mode. Full-scan spectra (m/z 120–1200) were acquired at a resolution of 30,000 (FWHM at m/z 400). Data-dependent MS/MS fragmentation was performed on the four most intense ions using a normalized collision energy of 55 eV, isolation width of 2 Da, and a resolution of 15,000.

Raw data were processed using Compound Discoverer (v3.3.1.111). A database of potential phlorotannins (DP 2–20) was generated in silico based on known polymerization patterns. Feature detection was performed with a mass tolerance of 5 ppm, minimum intensity threshold of 3000, and signal-to-noise ratio ≥ 3. Detected features were matched against the generated database and screened for characteristic neutral losses associated with phlorotannins. Common adducts were considered during annotation, and features were aligned across samples based on mass accuracy and retention time.

Putative phlorotannins were assigned based on accurate mass matching and fragmentation behavior, and results were exported for further analysis.

Supplementary Results

Chemical characzerization

Table S1. Total phenolic content (TPC), total polysaccharide content (TPSC), and antioxidant capacity (TEAC and FRAP) of AN and FV. TPC is expressed as mg phloroglucinol equivalents (PGE) per g dry weight, TPSC as mg glucose equivalents (GE) per g dry weight, and antioxidant capacity as µmol Trolox equivalents (TE) per g dry weight. Measurements were performed using photometric assays. Data adapted from [7].

	AN	FV
**TPC (mg PGE/g)**	44.09	92.05
**TPSC (mg GE/g)**	70.62	48.37
**TEAC (µmol TE/g)**	161.08	376.03
**FRAP (µmol TE/g)**	66.02	170.88

Table S2. Tentatively identified phlorotannins (PTs) in AN, including molecular formula, calculated molecular weight (MW), detected m/z, retention time (RT), degree of polymerization (DP), MS^2 information, and reference ion. Data adapted from [7].

	DP	Calculated MW (Da)	Formula	*m/z*	RT [min]	MS^2	Reference Ion
PT 1	2	250.05	$C_{12}H_{10}O_6$	249.04	2.95	No MS^2	$[M-H]^-$
PT 2	3	374.06	$C_{18}H_{14}O_9$	373.06	6.16	No MS^2	$[M-H]^-$
PT 3	4	498.08	$C_{24}H_{18}O_{12}$	497.07	3.70	No MS^2	$[M-H]^-$

https://doi.org/10.3390/md24050182

PT 4	4	498.08	$C_{24}H_{18}O_{12}$	497.07	9.42	No MS^2	$[M-H]^-$
PT 5	4	498.08	$C_{24}H_{18}O_{12}$	497.07	3.49	No MS^2	$[M-H]^-$
PT 6	4	498.08	$C_{24}H_{18}O_{12}$	497.07	1.81	No MS^2	$[M-H]^-$
PT 7	5	622.09	$C_{30}H_{22}O_{15}$	621.09	7.13	No MS^2	$[M-H]^-$
PT 8	5	622.10	$C_{30}H_{22}O_{15}$	621.09	2.99	No MS^2	$[M-H]^-$
PT 9	6	746.11	$C_{36}H_{26}O_{18}$	745.10	10.27	No MS^2	$[M-H]^-$
PT 10	7	870.13	$C_{42}H_{30}O_{21}$	869.12	11.41	DDA for preferred ion	$[M-H]^-$

Table S3. Tentatively identified phlorotannins (PTs) in FV, including molecular formula, calculated molecular weight (MW), detected m/z, retention time (RT), degree of polymerization (DP), MS^2 information, and reference ion. Data adapted from [7].

	DP	Calculated MW (Da)	Formula	*m/z*	RT [min]	MS^2	Reference Ion
PT 1	2	250.05	$C_{12}H_{10}O_6$	249.04	1.42	No MS^2	$[M-H]^-$
PT 2	3	370.03	$C_{18}H_{10}O_9$	369.02	13.45	No MS^2	$[M-H]^-$
PT 3	3	374.06	$C_{18}H_{14}O_9$	373.06	1.41	DDA for preferred ion	$[M-H]^-$
PT 4	3	374.06	$C_{18}H_{14}O_9$	373.06	2.19	DDA for preferred ion	$[M-H]^-$
PT 5	3	374.06	$C_{18}H_{14}O_9$	373.06	6.15	No MS^2	$[M-H]^-$
PT 6	4	498.08	$C_{24}H_{18}O_{12}$	497.07	9.26	No MS^2	$[M-H]^-$
PT 7	4	498.08	$C_{24}H_{18}O_{12}$	497.07	2.01	DDA for preferred ion	$[M-H]^-$
PT 8	4	498.08	$C_{24}H_{18}O_{12}$	497.07	1.78	No MS^2	$[M-H]^-$
PT 9	4	498.08	$C_{24}H_{18}O_{12}$	497.07	1.33	No MS^2	$[M-H]^-$
PT 10	5	1242.17	$C_{60}H_{42}O_{30}$	620.08	10.73	No MS^2	$[M-2H]^{2-}$
PT 11	5	622.09	$C_{30}H_{22}O_{15}$	621.09	2.96	DDA for preferred ion	$[M-H]^-$
PT 12	5	622.09	$C_{30}H_{22}O_{15}$	621.09	4.09	No MS^2	$[M-H]^-$
PT 13	5	622.10	$C_{30}H_{22}O_{15}$	621.09	11.46	No MS^2	$[M-H]^-$
PT 14	5	622.10	$C_{30}H_{22}O_{15}$	621.09	2.17	No MS^2	$[M-H]^-$
PT 15	5	622.10	$C_{30}H_{22}O_{15}$	621.09	1.92	No MS^2	$[M-H]^-$
PT 16	6	746.11	$C_{36}H_{26}O_{18}$	745.10	12.80	No MS^2	$[M-H]^-$
PT 17	6	746.11	$C_{36}H_{26}O_{18}$	745.10	12.57	No MS^2	$[M-H]^-$
PT 18	6	746.11	$C_{36}H_{26}O_{18}$	745.10	6.69	DDA for preferred ion	$[M-H]^-$
PT 19	6	746.11	$C_{36}H_{26}O_{18}$	745.10	3.19	No MS^2	$[M-H]^-$
PT 20	6	746.11	$C_{36}H_{26}O_{18}$	745.10	5.19	No MS^2	$[M-H]^-$
PT 21	6	746.11	$C_{36}H_{26}O_{18}$	745.10	3.77	No MS^2	$[M-H]^-$
PT 22	6	746.11	$C_{36}H_{26}O_{18}$	745.10	5.58	No MS^2	$[M-H]^-$
PT 23	6	746.11	$C_{36}H_{26}O_{18}$	745.10	2.57	No MS^2	$[M-H]^-$
PT 24	7	870.13	$C_{42}H_{30}O_{21}$	869.12	6.05	No MS^2	$[M-H]^-$
PT 25	7	870.13	$C_{42}H_{30}O_{21}$	869.12	9.69	DDA for preferred ion	$[M-H]^-$
PT 26	7	870.13	$C_{42}H_{30}O_{21}$	869.12	12.02	No MS^2	$[M-H]^-$
PT 27	7	870.13	$C_{42}H_{30}O_{21}$	869.12	9.39	DDA for preferred ion	$[M-H]^-$
PT 28	7	870.13	$C_{42}H_{30}O_{21}$	869.12	6.96	No MS^2	$[M-H]^-$
PT 29	8	994.14	$C_{48}H_{34}O_{24}$	993.13	11.07	DDA for preferred ion	$[M-H]^-$
PT 30	8	994.14	$C_{48}H_{34}O_{24}$	993.14	12.37	DDA for preferred ion	$[M-H]^-$
PT 31	8	994.14	$C_{48}H_{34}O_{24}$	993.14	10.30	No MS^2	$[M-H]^-$
PT 32	8	994.14	$C_{48}H_{34}O_{24}$	993.14	2.54	No MS^2	$[M-H]^-$
PT 33	9	1118.16	$C_{54}H_{38}O_{27}$	1117.15	13.14	No MS^2	$[M-H]^-$

https://doi.org/10.3390/md24050182

NF-κB signaling in HEK-Blue reporter cells

Table S4. AN and FV do not affect NF-κB activation in HEK-Blue null2 cells under basal conditions or following stimulation with LPS. Active SEAP levels in non-TLR4-expressing HEK-Blue null2 cells following a 24-h treatment with 200 µg AN or FV and/or 10 ng/ml LPS.

	HEK-Blue null2 cells SEAP/NF-κB activity OD620 values, background-corrected					
	**Control**	**AN 200 µg/g**	**FV 200 µg/g**	**LPS 10 ng/ml**	**LPS + AN 200 µg/g**	**LPS + FV 200 µg/g**
**Experiment 1**	0	0.004	0.002	0	0.014	0.013
	0	0.009	0.001	0.007	0.007	0.015
	0.009	0.006	0.001	0.003	0.004	0.006
**Experiment 2**	0.001	0	0	0	0.001	0
	0	0.002	0	0.002	0.001	0
	0.002	0.002	0	0.002	0.004	0.005
**Experiment 3**	0.008	0.005	0.004	0.004	0.009	0.011
	0.008	0.008	0.009	0.006	0.003	0.004
	0.008	0.008	0.008	0.01	0.008	0.005
**Average**	**0.0040**	**0.0049**	**0.0028**	**0.0038**	**0.0057**	**0.0066**
SD	0.0041	0.0031	0.0035	0.0033	0.0042	0.0054

Bacterial infection in D. melanogaster

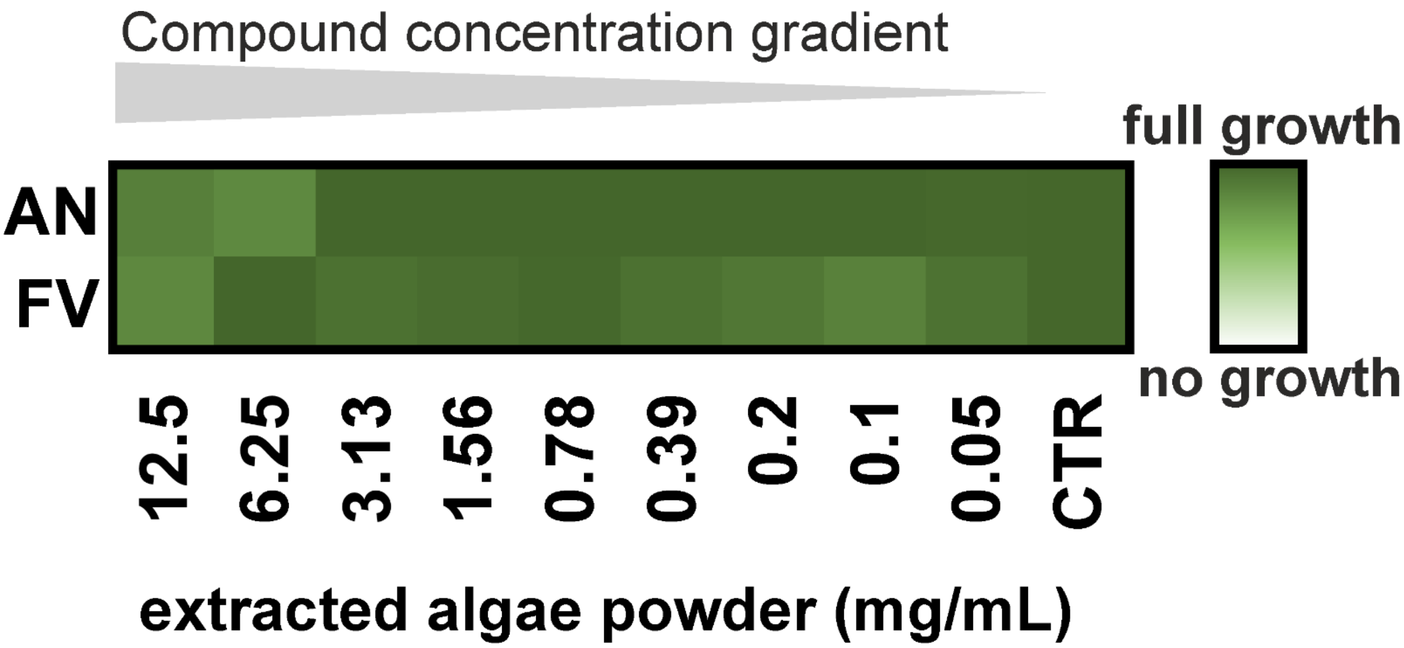

Figure S1. AN and FV do not alter bacterial growth at the tested concentrations. The antibacterial performance of AN and FV was tested with the broth microdilution method according to the Clinical and Laboratory Standards Institute (CLSI) [8].

https://doi.org/10.3390/md24050182

References

1. Singleton, V.L.; Orthofer, R.; Lamuela-Raventós, R.M. [14] Analysis of total phenols and other oxidation substrates and antioxidants by means of folin-ciocalteu reagent. *Methods in Enzymology : Oxidants and Antioxidants Part A*; Academic Press, 1999; pp 152–178, ISBN 0076-6879.
2. Wang, Y.; Jing, Y.; Leng, F.; Wang, S.; Wang, F.; Zhuang, Y.; Liu, X.; Wang, X.; Ma, X. Establishment and Application of a Method for Rapid Determination of Total Sugar Content Based on Colorimetric Microplate. *Sugar Tech* **2017**, *19*, 424–431, doi:10.1007/s12355-016-0473-7.
3. Miller, N.J.; Rice-Evans, C.; Davies, M.J.; Gopinathan, V.; Milner, A. A novel method for measuring antioxidant capacity and its application to monitoring the antioxidant status in premature neonates. *Clinical science (London, England : 1979)* **1993**, *84*, 407–412, doi:10.1042/cs0840407.
4. Rice-Evans, C.; Miller, N.J. Total antioxidant status in plasma and body fluids. *Methods in enzymology* **1994**, *234*, 279–293, doi:10.1016/0076-6879(94)34095-1.
5. Re, R.; Pellegrini, N.; Proteggente, A.; Pannala, A.; Yang, M.; Rice-Evans, C. Antioxidant activity applying an improved ABTS radical cation decolorization assay. *Free Radical Biology and Medicine* **1999**, *26*, 1231–1237, doi:10.1016/s0891-5849(98)00315-3.
6. Stratil, P.; Klejdus, B.; Kubán, V. Determination of total content of phenolic compounds and their antioxidant activity in vegetables--evaluation of spectrophotometric methods. *Journal of agricultural and food chemistry* **2006**, *54*, 607–616, doi:10.1021/jf052334j.
7. Karlsberger, L.; Sandner, G.; Molčanová, L.; Rýpar, T.; Ladirat, S.; Weghuber, J. Antioxidant Power of Brown Algae: Ascophyllum nodosum and Fucus vesiculosus Extracts Mitigate Oxidative Stress In Vitro and In Vivo. *Mar. Drugs* **2025**, *23*, doi:10.3390/md23080322.
8. Barry, A.L. *Methods for determining bactericidal activity of antimicrobial agents : approved guideline / Arthur L. Barry [and 5 others];* National Committee for Clinical Laboratory Standards: Wayne, PA, 1999, ISBN 1562383841.

Disclaimer/Publisher's Note: The statements, opinions and data contained in all publications are solely those of the individual author(s) and contributor(s) and not of MDPI and/or the editor(s). MDPI and/or the editor(s) disclaim responsibility for any injury to people or property resulting from any ideas, methods, instructions or products referred to in the content.
